# A review of lactate-lactylation in malignancy: its potential in immunotherapy

**DOI:** 10.3389/fimmu.2024.1384948

**Published:** 2024-05-08

**Authors:** Jinhui Zha, Junan Zhang, Jingfen Lu, Guangcheng Zhang, Mengzhan Hua, Weiming Guo, Jing Yang, Gang Fan

**Affiliations:** ^1^ Department of Urology, Huazhong University of Science and Technology Union Shenzhen Hospital, Shenzhen, China; ^2^ Department of General Surgery, Shenzhen University General Hospital, Shenzhen, China; ^3^ Department of Basic Medicine, Shenzhen University, Shenzhen, China; ^4^ The First Clinical Medical College, Guangzhou University of Chinese Medicine, Guangzhou, China; ^5^ Department of Sports Medicine Huazhong University of Science and Technology Union Shenzhen Hospital, Shenzhen, China; ^6^ Endocrinology Department, Huazhong University of Science and Technology Union Shenzhen Hospital, Shenzhen, China

**Keywords:** lactate, lactylation, metabolic reprogramming, tumor immunotherapy, microenvironment

## Abstract

Lactic acid was formerly regarded as a byproduct of metabolism. However, extensive investigations into the intricacies of cancer development have revealed its significant contributions to tumor growth, migration, and invasion. Post-translational modifications involving lactate have been widely observed in histone and non-histone proteins, and these modifications play a crucial role in regulating gene expression by covalently attaching lactoyl groups to lysine residues in proteins. This discovery has greatly enhanced our comprehension of lactic acid’s involvement in disease pathogenesis. In this article, we provide a comprehensive review of the intricate relationship between lactate and tumor immunity, the occurrence of lactylation in malignant tumors, and the exploitation of targeted lactate-lactylation in tumor immunotherapy. Additionally, we discuss future research directions, aiming to offer novel insights that could inform the investigation, diagnosis, and treatment of related diseases.

## 1 Introduction

**Figure 1:**
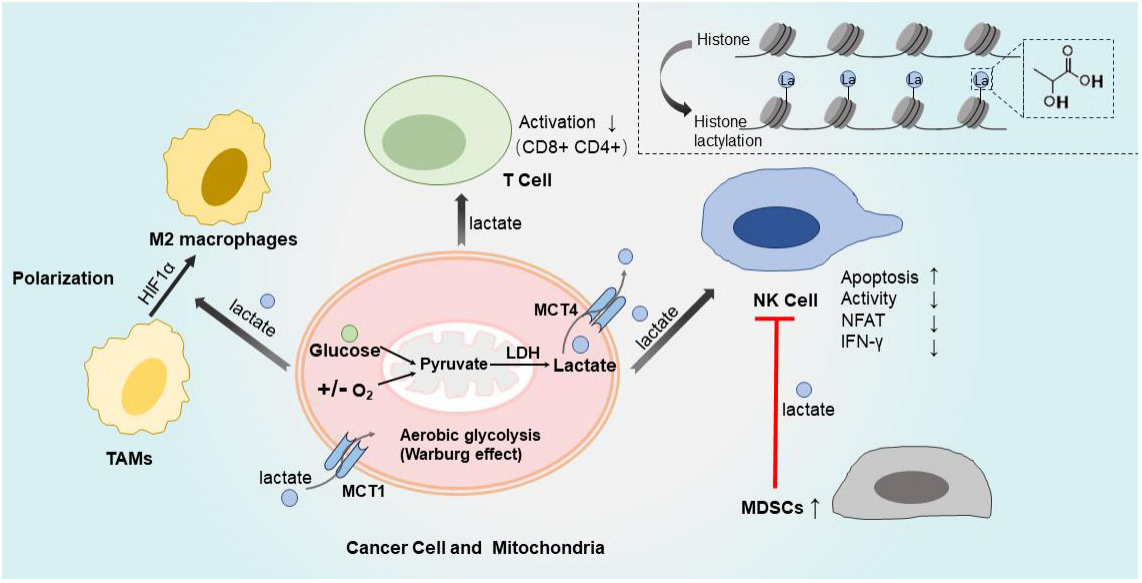
Lactate and tumor immune microenvironment. Cancer cells produced significant amounts of lactate via glycolysis, irrespective of the presence of oxygen, which is called Warburg effect. During the process of glycolysis, pyruvate conversion to lactate through the action of LDH. Lactate are export by MCT4 from cytoplasm to extracellular fluid, MCT1 import lactate to cytoplasm. Then different kinds of immune cells are influenced by the TME of high levels of lactate, with suppression of anti-tumor immune responses. The activation of effector CD8+ and CD4+ T cells is suppressed when the pH value decreased because lactate increased. High levels of lactate directly impede the activity of NK cells and induce apoptosis. Additionally, lactate inhibits the activation of NFAT in NK cells, resulting in reduced production of IFN-γ. Moreover, lactate indirectly suppresses NK cells by increasing the population of MDSCs.HIF1α promotes tumor growth and facilitates TAMs transformation into M2-like phenotype, which is induced by lactic acid derived from tumors. The top right corner represents the process of histone lactylation modification. LDH, lactate dehydrogenase; MCT1/4, monocarboxylate transporter 1/4; MDSCs, myelid derived suppressor cells; TAMs, transformation of tumor-associated macrophages; NFAT, nuclear factor-activated T cells; TME, tumor microenvironment; IFN-γ, Interferon γ.

During the process of glycolysis, pyruvate molecules are converted into lactate through the action of cytoplasmic lactate dehydrogenase (LDH), rather than directly entering the tricarboxylic acid (TCA) cycle (1). In 1923, Otto Heinrich Warburg made the observation that cancer cells exhibit a proclivity for producing significant amounts of lactate via glycolysis, irrespective of the presence of oxygen (**Figure 1**). This observation came to be known as the Warburg effect (2). Subsequent investigations have revealed that lactate serves as a signaling molecule, exerting notable influences on immune cell function, immune response modulation, cell metabolism regulation, and immune surveillance (3–5). The tumor microenvironment (TME) constitutes a multifaceted network comprising tumor cells, stromal cells, blood vessels, endothelial cells, growth factors, nutrients, and cell metabolites (6). Expanding upon the postulated Warburg effect hypothesis, researchers have observed that the release of lactate from tumor cells contributes to the acidification of the TME. This acidic microenvironment promotes tumor angiogenesis, triggers metastasis development, induces drug resistance, and facilitates immune evasion (7). Recent studies have additionally indicated that cancer cells can utilize lactate as an energy source (8, 9). Consequently, therapeutic strategies targeting metabolic processes, including lactate synthesis, have emerged as potential innovative approaches for the treatment of cancer patients (10).

Lactylation, alternatively known as lysine lactylation (Kla), is a post-translational modification (PTM) that involves the covalent attachment of lactic acid moieties to protein lysine residues, thereby exerting influence on gene expression regulations. The elucidation of lactylation has significantly broadened our comprehension of lactate’s role in biological systems. Consequently, the presence of lactylated histone and non-histone proteins holds paramount importance in the modulation of gene transcription (11). As a prevalent PTM, lactate-induced protein lactylation not only contributes to normal physiological processes (12), such as the regulation of immune homeostasis during cardiac repair (13), but also plays a significant role in the etiology and progression of various diseases, particularly cancer (14, 15). Evidence suggests that lactylation of tumor cells, tumor stem cells, and tumor-infiltrating immune cells in the TME can actively contribute to cancer progression through downstream modulation of gene expression, thus emerging as a promising therapeutic target in cancer treatment (16). However, our understanding of the intricate regulatory mechanisms involving lactate-induced lactylation in malignant tumors and the clinical potential of therapeutic interventions targeting this pathway remains incomplete.

Here, we have summarized recent literature in this area to gain a more encompassing understanding on the current research landscape, delineate potential avenues for future investigation, overcome the constraints of current cancer treatments, and present novel avenues for therapeutic strategies targeting lactate-induced lactylation.

## 2 Lactate and tumor immune microenvironment

The ability of cancer cells to undergo metabolic reprogramming and avoid detection by the immune system is regarded as an emerging hallmark of cancer (17). As previously mentioned, the Warburg effect is a pivotal aspect of energy metabolism in cancer cells, where they preferentially rely on glycolysis to sustain biosynthetic processes (18). This results in the production of high levels of lactate, actively maintaining an acidic TME that suppresses anti-tumor immune responses (19). Consequently, lactate plays a crucial role in bridging metabolic reprogramming with immune evasion mechanisms (20). Remarkably, lactate has intricate effects on both tumor cells and immune cells that infiltrate the tumor within the TME (**Figure 1** and **Table 1**).

**Table 1:** Lactate-Lactylation in Malignancy and treatment.

Malignancy	Objects	Intervention	Comments	Ref
Lactate and tumor immune microenvironment				
Neuroblastoma	Cell lines	100% O_2_ or N_2_	Warburg effect contribute to cellular lactic acid production.	(19)
Melanoma	Cell lines, mouse	MCT1 inhibitor	Treg cell specific deletion of MCT1 not only results in decreased tumor growth but synergy with checkpoint blockade immunotherapy.	(21)
Melanoma	Cell lines; mouse; human samples	LDH-A ^low^; Lactate treatment	Increased lactic acid inhibits tumor immunosurveillance and promoting tumor growth.	(22)
Pancreatic cancer	Cell lines, mouse	LDH-A-deficient; Lactate treatment	Lactate inhibits NK cell function via direct inhibition of cytolytic function as well as indirectly by increasing the numbers of MDSCs.	(23)
Lung carcinoma; lung carcinoma; colon carcinoma	Cell lines, mouse	Hypoxia; HIF1a ^-/-^	Lactic acid has a critical function in signaling, mediated by HIF1a, through inducing the M2-like polarization.	(24)
Breast cancer	Cell lines, mouse	Gpr132-KO; oxamic acid	Lactate activated M2-like macrophage, facilitates cancer cell adhesion, migration, and invasion.	(25)
Lysine lactylation in malignancy				
Non-small cell lung cancer	Cell lines, human samples	Lactate stock solution	Lactate modulates cellular metabolism through histone lactylation-mediated gene expression.	(26)
Hepatocellular carcinoma	Cell lines; human samples	Lactylome profiling; lactate treatment	Lactylation at K28 facilitates the proliferation and metastasis of hepatocellular carcinoma cells.	(27)
Glioblastoma	Cell lines, mouse, human samples	Bioinformatics analysis; Xenograft	NF-κB pathway promoted Warburg Effect, induced the lactylation of H3 histone associating with poor progression of glioblastoma.	(28)
Clear cell renal cell carcinoma	Cell lines; mouse; human samples	Xenograft; oxamate	PDGFRβ signaling is shown to stimulate histone lactylation, thereby forming an oncogenic positive feedback loop in ccRCC.	(29)
Prostate cancer	Cell lines; tissue microarray	Lactate treatment; silencing of KIAA1199	Lactate is transcriptional enhancer of KIAA1199. Silencing of KIAA1199 inhibited angiogenesis and VM in pca.	(30)
Pancreatic ductal adenocarcino-ma	Cell lines, mouse; human tissue	NUSAP1 treatment	NUSAP1 plays a critical role in metastasis of PDAC by regulating lactate dehydrogenase A mediated glycolysis.	(31)
Melanoma	Cell lines, mouse, human tissues	lactylation inhibitors	Histone lactylation contributes to tumorigenesis by facilitating YTHDF2 expressio-n.	(32)
Melanoma	Cell lines, mouse, human tissues	Xenograft; ALKBH3; lactylation inhibitors(oxamate and 2-DG)	Histone lactylation increases the expression of ALKBH3 thereby accelerating tumor.	(33)
Colorectal cancer	Cell lines, mouse, human tissues	Xenograft; glycolytic inhibitors (oxamate and 2-DG); LDH-A; Bevacizumab	CRC patients resistant to bevacizumab presented with elevated levels of lactylation.	(34)
Colon cancer	Cell lines, mouse, human tissues	Xenograft; target to lactylation of MRE11	Inhibition of CBP or LDH downregulated lactylation of MRE11 and enhanced chemosensitivity of tumor cells.	(35)
Gastric cancer	Cells lines, mouse, human tissues	Xenograft; copper stress; deacetylation enzyme	Elevated METTL lactylation improves the therapeutic efficacy of the copper ionophore elesclomol.	(36)
Neuroblastoma	Cells lines	Deacetylation enzyme (SIRT2)	As an efficient inhibition for multiple histone lactylation sites of histones in neuroblastoma cells.	(37)
Acute myeloid leukemia	Cell lines, human blood	Upregulated glycolysis (STAT5)	The accumulation of lactate driven by facilitated histone lactylation on PD-L1 promoter and ultimately induced PD-L1 expression.	(38)
Bladder cancer	Cell lines, mouse, human tissues	Overexpression of circXRN2 (transfect plasmids)	CircXRN2 suppresses tumor progression driven by H3K18 lactylation.	(39)
Lactate-Lactylation in Malignancy treatment				
MCT1-targeted treatment				
Advanced solid tumors or lymphoma	Human (Phase I trial)	MCT1 inhibitor	AZD3965 is tolerated, the dose-limiting toxicities were on target and dose-dependent. A Phase 2 dose of 10 mg was established.	(40)
PD-1 & MCT1/4				
MYC-amplified tumors and liver tumors	Cell lines, mouse, human and human tissues	MCT1; highly glycolytic; Anti-PD-1 mAb RMP1-14 or nivolumab	Treg cells actively absorbed LA through MCT1, enhancing the expression of PD-1, and dampening expression of PD-1 by effector T cells.	(41)
Melanoma	Cell lines, mouse, human tissues	m^6^A demethylases; anti–PD 1 pembrolizumab and nivolumab	Alkbh5 modulates Mct4/Slc16a3 expression, lactate content and the composition of tumor-infiltrating Treg and myeloid derived suppressor cells.	(42)
Hepatocellular carcinoma	Mouse; human tissues	MCT4 inhibition; anti–PD 1 toripalimab	Inhibition of MCT4 can heighten activity of CD8+ T cells and reduce acidification in tumor microenvironment.	(43)
Colorectal carcinoma	Cell lines, mouse; human blood	MCT4 inhibition; anti-PD-L1 antibody	Combination of MCT4 and ICB increased intratumoral pH, delayed tumor growth, and prolonged survival *in vivo*.	(44)
PD-1 & LDH-A				
Non-small cell lung cancer	Mouse	Oxamate; anti–PD 1 pembrolizumab	Preclinical findings: LDH inhibitor oxamate treatment enhanced the therapeutic effects of pembrolizumab.	(45)
Melanoma	Cell Lines; mouse	Deletion of LDH-A; Anti-PD-1 antibody (clone 29F.1A12)	Deficiency of LDH-A increased infiltration of NK cells and CD8+ cytotoxic T cells, improving the efficacy of anti-PD-1 therapy.	(46)
Cancer vaccines				
Melanoma and colon adenocarcinoma	Cell lines, mouse, Human blood	Glucose or sodium lactate; CD8+ T cellvaccine	HDAC inhibition induced by lactate enhanced CD8+ T cell exhaustion efficiently inhibit tumor growth.	(47)
Lymphoma	Cell lines, mouse	Lactic acid; irradiation	Lactic acid could augment the immunogenicity of whole UV-irradiated tumor cell vaccines.	(48)
CAR-T therapy				
Glioblastoma	Cell lines, mouse	Oxamate,LDH-A inhibitor; CAR-T cells	Oxamate promoted immune activation of tumor-infiltrating CAR-T cells.	(49)

YTHDF2, YTH N6-methyladenosine RNA-binding protein 2; CRC, colorectal cancer; LDH, lactate dehydrogenase; HR,homologous recombination; PD-1/PD-L1, Programmed cell death protein 1/programmed cell death-ligand; MCT1/4, monocarboxylate transporter 1/4; LA,lactic acid; ROS, Reactive Oxygen Species; NF-κ, nuclear factor kappa-B;ICB, immune checkpoint blockade; DC, dendritic cell; MDSC, Myeloid-derived suppressor cells; CAR-T,chimeric antigen receptor T cell; NSCLC, non-small-cell lung cancer; ccRCC, clear cell renal cell carcinoma; VHL, Inactive von Hippel-Lindau; PDGFRβ, platelet-derived growth factor receptor β; HIF1α,hypoxia-inducible factor 1α. NUSAP1, Nucleolar and spindle associated protein 1; PDAC, pancreatic ductal adenocarcinoma; Gpr132,G protein-coupled receptor 132;KO/^-/-^,knock out.

Excessive lactate within the TME can hinder the effectiveness of anti-tumor immunity by interfering with the function of various immune cells that infiltrate the tumor (50). Watson MJ, et al., and Angelin, Alessia et al. (21, 51) have confirmed that the activation of effector CD8+ and CD4+ T cells is commonly suppressed when the pH of the TME falls within the range of 6.0 to 6.5, resulting in diminished cytotoxicity and cytokine production. Lactic acid plays a crucial role in enhancing the growth and performance of tumor-infiltrating regulatory T cells (Tregs). Kouidhi S, et al. (52) and Wu H, et al. (53) have demonstrated that the reversal of the acidic TME through the application of proton pump inhibitors can restore the inhibition of anti-tumor immunity and enhance immunotherapy, thereby corroborating these findings. Moreover, a number of studies have indicated that a high concentration of lactate can impede the activity of natural killer (NK) cells and induce apoptosis in these cells (54–56). Mechanistically, Brand A, et al. (22) revealed that lactic acid impedes the activation of nuclear factor-activated T cells (NFAT) in NK cells, resulting in reduced production of IFN-γ. Husain Z, et al. (23) discovered that lactate not only directly impairs the functionality of NK cells, but also indirectly suppresses these cells by increasing the population of myeloid-derived suppressor cells (MDSCs) (**Figure 1**).

In a recent study by Colegio OR et al. (24), it was discovered that lactic acid derived from tumors plays a crucial role in inducing the transformation of tumor-associated macrophages (TAMs) into an M2-like phenotype. This process is facilitated by the activation of hypoxia-inducing factor 1α (HIF1α), which subsequently promotes tumor growth within the context of the TME (**Figure 1**). Significantly, the regulation of extracellular signals also plays a crucial role in several intracellular signaling pathways, a mechanism that holds particular importance within TME (57). Consistent with this, Chen P. et al. (25, 58) demonstrated that lactate induces the polarization of M2 macrophages through the upregulation of vascular endothelial growth factor (VEGF) and arginase-1 (ARG1) via the extracellular signal-regulated kinase/transcription 3 (ERK/STAT3) signaling pathway.

## 3 Lysine lactylation in malignancy

As a ubiquitous biological process, lactylation has been proven to be associated with the growth of numerous cancers. Recent investigations have not just delved into its crucial role in ocular melanoma, colorectal cancer, gastric cancer, acute myeloid leukemia, and bladder cancer (details below), but also investigated its implications in non-small cell lung cancer (26), hepatocellular carcinoma (27), glioma (28), clear cell renal cell carcinoma (29), prostate cancer (30), and pancreatic ductal adenocarcinoma (31) (**Table 1**). In a recent investigation involving 82 cases of ocular melanoma and 28 cases of normal tissues, researchers observed elevated levels of lactylation in tumor tissues compared to normal tissues, particularly at the histone H3K18 site. This process was found to hinder the proliferation and migration of tumor cells (32). Mechanistically, lactylation of H3K18 affects the development of ocular melanoma by regulating the reader protein YTHDF2, which is responsible for RNA m6A modifications. Notably, increased expression of YTHDF2 is associated with a negative prognosis for patients (59). Additional research has unveiled that histone lactylation increases the expression of ALKBH3 in ocular melanoma patients at high risk. This modification influences the formation of the tumor suppressor protein PML condensate by reducing N1-methyladenosine (m1A) methylation on SP100A, thereby accelerating tumor progression (33). Thus, strategies targeting ALKBH3 may offer substantial potential for melanoma treatment.

Chemotherapeutics, including platinum drugs and targeted agents such as bevacizumab, play essential roles in the management of advanced and metastatic colorectal cancer (CRC) (60, 61). Nevertheless, the widespread issue of drug resistance cannot be overlooked (62–64). Notably, CRC patients who are resistant to bevacizumab therapy exhibit significantly elevated glycolytic signaling and histone H3K18la (histone H3 lysine-18 lactylation) levels. These observations may provide insight into a potential underlying cause for patient resistance to this agent (34). In a separate study, investigators explored organoid models and xenotransplantation models (PDXs) of CRC patients, revealing that the Warburg effect can enhance homologous recombination (HR) and therefore contribute to chemotherapy resistance in cancer cells. Additionally, they observed that the inhibition of HR and reversal of drug resistance can be achieved by using cell-penetrating peptides that block the lactylation of MRE11, which encodes a nuclear protein involved in HR and DNA double-strand break (DSB) repair. Consequently, this approach increases the sensitivity of cancer cells to cisplatin and polyADP ribose polymerase inhibitors (PARPi) (35). This finding exposes the critical regulatory role of MRE11 lactylation in HR and offers a novel perspective on the relationship between tumor cell metabolism and DSB. Furthermore, it suggests a potential therapeutic strategy for overcoming chemotherapy resistance in CRC patients (65).

Elevated lactate and copper concentrations have been observed in gastric cancer (GC) (36). The researchers discovered that the m6A modification on ferredoxin 1 (FDX1) mRNA, mediated by an atypical methyltransferase called METTL16, plays a crucial role in copper-induced apoptosis. To further clarify, FDX1 encodes a reductase responsible for reducing Cu^2+^ to its more toxic form, Cu^1+^. They found that under conditions of copper stress, the lactylation of METTL16 at the K229 site is enhanced but inhibited by SIRT2 (37). Interestingly, the elevated levels of lactylation induced by METTL16 can enhance the therapeutic effectiveness of the copper ionophore elesclomol (66). When elesclomol is combined with the SIRT2 inhibitor AGK2, it induces copper-induced apoptosis in gastric tumors both *in vitro* and *in vivo* (36). This combination therapy offers a promising treatment strategy for GC.

In acute myeloid leukemia (AML), the upregulation of glycolysis by STAT5 results in the accumulation of lactate (38). This, in turn, promotes the translocation of E3 binding protein (E3BP) and histone lactylation to the nucleus, ultimately enhancing the transcription of PD-L1 in leukemia cells. The inhibition of PD-1/PD-L1 using immune checkpoint inhibitors (ICIs) can restore the activity of CD8+ T cells when co-cultured with AML cells that express high levels of STAT5. This suggests that PD-1/PD-L1 based immunotherapy may be beneficial for AML patients with STAT5-induced glycolysis and lactate accumulation (45, 67, 68).

A comprehensive investigation has been conducted to gain a deeper understanding of the underlying mechanism by which circXRN2 regulates tumor growth in bladder cancer (39). The findings revealed that circXRN2 has the capacity to bind with LATS1 protein, thus protecting it from undergoing speckle-type POZ protein-mediated ubiquitination and subsequent degradation. This interplay triggers activation of the Hippo signaling pathway, consequently restraining H3K18 lactylation and ultimately impeding the progression of bladder cancer. Importantly, these groundbreaking observations shed light on a potentially robust target for therapeutic intervention in the clinical management of bladder cancer.

## 4 Targeted lactate-lactylation in tumor immunotherapy

### 4.1 Targeted lactate-lactylation in combination with immune checkpoint inhibitor therapy

ICIs, as a revolutionary breakthrough in tumor immunotherapy, have demonstrated remarkable efficacy and long-lasting therapeutic responses in a subset of tumor patients (69–71). Currently FDA-approved ICIs encompass diverse formulations targeting programmed cell death 1 (PD-1), programmed cell death ligand 1 (PD-L1), and cytotoxic T lymphocyte-associated antigen-4 (CTLA-4) (72).

However, up to 85% of tumor patients exhibit poor response to ICIs. This can be attributed to individual genetic variations and the unique metabolic landscape of the TME (73, 74). Notably, the TME serves as one of the key contributing factors to this phenomenon (75, 76). In line with this notion, synergistic effects have been observed when combining mTOR inhibitors with glycolysis inhibitors across various cancer types including lymphoma, leukemia, and colorectal cancer (77, 78). Therefore, exploring metabolic modulators within the TME as adjuvants for combination therapy involving ICIs holds great promise (**Table 1**).

Kumagai et al. (41) recently reported that in highly glycolytic TME conditions, such as MYC-amplified tumors and liver tumors, Tregs uptake lactic acid via monocarboxylate transporter 1 (MCT1), which enhances nuclear translocation of NFAT1 and promotes PD-1 expression. Consequently, targeting PD-1 activation alone may lead to treatment failure due to the activation of PD-1+ Treg cells. This observation highlights the potential role of lactic acid as an effective checkpoint in regulating Treg function under low glucose conditions, and further supports the theoretical basis for synergistic effects attained by combining ICIs with strategies that target lactic acid metabolism.

It has been previously observed by other researchers that inhibiting or eliminating the m^6^A demethylase ALK-BH5 during anti-PD-1 therapy in mouse models of melanoma and colorectal cancer leads to a notable decrease in lactate levels within the TME. Simultaneously, it also reduces the recruitment of Treg cells and myeloid-derived suppressor cells (MDSCs). These observations emphasize the potential of ALK-BH5 inhibitors as a novel approach to tackling resistance to tumor ICIs (42, 79).

A recent study has shown that inhibiting the high-affinity lactate transporter MCT4, either genetically or pharmacologically (43, 80), greatly enhances the therapeutic efficacy of anti-PD-1 therapy. This improvement was observed in a mouse model of hepatocellular carcinoma (HCC), resulting in prolonged survival. This effect can potentially be attributed to the heightened activity of CD8+ T cells, a reduction in tumor microenvironment acidification, and the increased secretion of chemokine ligands (81). These outcomes were induced by the ROS/NF-κB signaling pathway. Furthermore, the research team discovered higher levels of MCT4 expression in HCC patients who did not respond well to toripalimab neoadjuvant therapy. Similarly, the combination treatment of MCT4 inhibitors and anti-PD-L1 therapy exhibited beneficial effects in 3D colorectal cancer sphere models. However, this positive outcome was not observed when combining MCT1 inhibitor AZD3965 (44) with anti-PD-L1 therapy. Notably, AZD 3965 is currently undergoing a dose-escalation Phase I trial for the treatment of advanced solid tumors and lymphomas (NCT 01791595) (40).

In addition, extensive research has focused on therapeutic strategies targeting LDH. It has been reported that targeting LDH to reduce the production of lactic acid can turn tumors into “hot” tumors, characterized by a high degree of T cell infiltration and a better response towards ICIs therapies (45, 82). Qiao, T et al. (45) demonstrated in a humanized mouse model of non-small cell lung cancer (NSCLC) that the LDH inhibitor oxamate may enhance the therapeutic effect of pembrolizumab by a mechanism mainly associated with an increase in activated CD8 + T cells in tumors. Consistent with this, other researchers have found that mice with lactate dehydrogenase A (LDH-A) deficient B16-F10 melanoma have a better response to anti-PD-1 treatment, which is manifested by increased infiltration of NK cells and CD8 + cytotoxic T cells (46). Interestingly, although it is also a glycolytic pathway inhibitor, it is different from proton pump inhibitors (83) because LDH-A is not a key enzyme in normal cell metabolism, selective targeting of LDH-A has minimal theoretical side effects, making it a new target with more promising prospects and development value (45).

### 4.2 Effects of lactate and lactic acid in cancer vaccines

As an active immunotherapy, tumor vaccines utilize tumor-specific antigens (TSAs) or tumor-associated antigens (TAAs) to stimulate the body’s specific immune response, which has emerged as a prominent area of research in tumor immunotherapy (84). However, the intricate immune evasion mechanisms employed by tumor tissues pose challenges for achieving desired efficacy with tumor vaccines (85), and inadequate immunogenicity remains a key concern in current clinical applications (86).

Numerous researchers have explored the impact of lactate and lactic acid on the effectiveness of tumor vaccines. Feng et al. (47) compared the therapeutic effects of PC7A nano-tumor vaccine in lactate solution (1.68 g/kg, pH 7.4) and glucose solution (5 g/kg, pH 7.4) using an MC38 mouse tumor model, revealing significantly improved anti-tumor efficacy in the lactate group. Conversely, decreased anti-tumor efficacy was observed in the glucose group. Notably, subcutaneous injection of sodium lactate did not elevate tumor acidity; instead, it solely augmented the lactate concentration. This suggests that the lactate’s positive effect on anti-tumor immunity is not necessarily tied to pH alterations but may potentially be attributed to the enhanced exhaustion of CD8+ T cells mediated by lactate-induced HDAC inhibition. These findings suggest that lactate may enhance the effectiveness of T cell-based immunotherapies such as tumor vaccines. Another study demonstrated that lactic acid can augment the immunogenicity of whole UV-irradiated tumor cell vaccines by promoting dendritic cell (DC) maturation and aggregation within mouse xenograft models while enhancing phagocytosis (48). Given DCs’ crucial role in anti-tumor immunity, it is speculated that lactic acid-stimulated tumor vaccines may be more effective at inducing immune responses (87). Additionally, increased numbers of IFN-γ-expressing CD4+T and CD8+T cells were detected within spleen and lymph nodes from experimental mice, indicating potential dominance of cellular immunity mediated by CD8+T cells during this process—consistent with previous studies’ conclusions (88). Furthermore, the injection of lactic acid-stimulated tumor vaccines significantly reduces the number of CD11b+Gr1+MDSCs in tumor tissues, which plays a crucial role in immune evasion, tumor occurrence, and development (89). The aforementioned studies collectively indicate that lactate and lactic acid may exhibit different effects on tumor cells and infiltrating immune cells *in vitro* compared to *in vivo* experiments. However, at high concentrations, they can induce tumor cell apoptosis and enhance the efficacy of tumor vaccines (48).

### 4.3 Lactate-lactylation in CAR-T therapy

In recent years, chimeric antigen receptor T cell (CAR-T) therapy has emerged as a promising immunotherapy for various hematological tumors due to its remarkable effectiveness (90, 91). Nevertheless, the therapeutic outcome of CAR-T therapy in solid tumors remains unsatisfactory due to limitations imposed by the immunosuppressive TME and other factors (92, 93).

Numerous researchers have attempted to investigate the impact of lactate-lactylation targeted strategies on the efficacy of tumor vaccines. Sun et al. conducted a study exploring combined treatment with an LDH-A inhibitor and CAR-T therapy in a mouse model of glioblastoma multiforme (GBM) (49). Their findings demonstrated that LDH-A inhibitor Oxamate effectively reduced CAR-Treg cell levels and adenosine production within the TME by decreasing histone H3K18 lactylation levels. This reduction downregulated CD39, CD73, CCR8 gene promoter activity while reprogramming glucose metabolism in tumor stem cells. Ultimately, it promoted immune activation within the TME and showcased potential for improving GBM patient prognosis when combined with CAR-T therapy (94, 95). Additionally, some scholars have proposed that lactate may exert an immunoprotective role against anti-tumor immunity. The addition of lactate during the ex vivo expansion of T cells could potentially enhance the efficacy of CAR-T therapy (47), further highlighting the complex effects of lactate on both tumors and immune cells.

## 5 Conclusion and perspective

When confronted with environmental changes, tumor cells undergo metabolic reprogramming to adapt to the new environment (96). Lactate, as a byproduct of glycolysis, can lactylate both histone and non-histone proteins under the influence of specific enzymes (11). Although lactate was once regarded as a mere “metabolic waste” of glycolysis, numerous studies have gradually unraveled the Warburg effect, confirming its integral role in the TME. It is involved in tumor angiogenesis and mediates immune suppression among other processes (7), making it a potential target for cancer therapy. Further exploration of lactate’s potential role in tumorigenesis and the immune microenvironment is expected to yield fascinating discoveries.

Based on these findings, targeting lactate-lactylation and its associated metabolic pathways has emerged as a novel research avenue for cancer therapy. One strategy involves interfering with tumor cell metabolism by inhibiting lactate production and transport to reduce lactate accumulation and immunosuppression within the TME. Another strategy focuses on developing targeted drugs that affect lactate-lactylation to interfere with its effects on tumors and immune cells. Currently, notable progress has been achieved in studies targeting MCT4 (43, 44) and LDH (45, 49), but inhibitors targeting glycolysis are still at the preclinical stage involving animal model experiments without sufficient clinical translation. Despite the potential of targeting Lactate-Lactylation, there exist several challenges and limitations that hinder its clinical translation. For instance, shared enzymes exist between lactylation and acetylation, posing the risk of complications during treatment. Moreover, the risk lies in the expression of MCT1 in normal tissues, particularly the retina and heart. There have been reports of reversible vision loss and elevations in cardiac troponin levels in patients undergoing MCT1-targeted therapies, which are indicators of retinal effects and myocardial injury, respectively (40). It is imperative to carefully consider the balance between potential benefits and risks when pursuing targeted lactate therapy and explore strategies to mitigate these side effects. Still, inhibitors with more specificity targeting MCT and LDH remains limited. On top of that, current strategies and clinical trials do not prioritize the consideration of pH value, an aspect that could significantly impact therapeutic outcomes.

Although current research has gradually illuminated the role of lactate-lactylation in the TME, there are still intriguing avenues to explore. Firstly, certain studies have indicated that the immunoprotective effect of lactate may be underestimated. In contrast to lactic acid, lactate might exert an immunoprotective role against tumor immunity, primarily due to the confounding influence of proton-induced immunosuppression within the acidic TME. This discovery offers a novel perspective for further investigation (47). Additionally, investigations into the impact of lactic acid and lactate on tumor cells and immune infiltrates within TME can sometimes be influenced by experimental conditions both *in vitro* and *in vivo* (48). Consequently, comprehending the effects of lactic acid and lactate on TME and tumor immunotherapy is likely intricate; thus necessitating additional reliable experimental studies to clarify their potential implications on TME while reassessing specific roles played by lactic acid and lactate.

Currently, the bulk of investigations on lactylation focus on its downstream. To fully understand the complex conditions that lead to lactylation, more researches are needed. Besides, the specific reader of lactylation remains unclear, and the study concerning inhibitors for lactylation epigenetic tools are limited. Notably, lactylation and acetylation share certain enzymes, indicating a potential competitive relationship. Thus, it becomes imperative to discern its complex interplay with other PTMs such as acetylation, methylation, ubiquitination, SUMOylation etc., within organisms; thereby further investigating whether lactylation exerts broader impacts on physiological and pathological processes within organisms.

To summarize, Lactate-Lactylation plays a pivotal role in tumor metabolic reprogramming as well as tumor immunity. Enhancing our understanding of the intricate involvement of lactate-lactylation in TME will facilitate better understanding of tumorigenesis and development biological processes. Consequently, this will pave the way for the exploration of novel therapeutic targets aimed at improving the prognosis of cancer patients.

## Author contributions

JZ: Conceptualization, Data curation, Formal analysis, Funding acquisition, Investigation, Methodology, Project administration, Resources, Software, Supervision, Validation, Visualization, Writing – original draft, Writing – review & editing. JZ: Conceptualization, Data curation, Formal analysis, Funding acquisition, Investigation, Methodology, Project administration, Resources, Software, Supervision, Validation, Visualization, Writing – original draft, Writing – review & editing. JL: Data curation, Formal analysis, Resources, Visualization, Software, Writing – original draft, Writing – review & editing. GZ: Conceptualization, Data curation, Formal analysis, Funding acquisition, Investigation, Methodology, Project administration, Resources, Software, Supervision, Validation, Visualization, Writing – original draft, Writing – review & editing. MH: Conceptualization, Data curation, Formal analysis, Funding acquisition, Investigation, Methodology, Project administration, Resources, Software, Supervision, Validation, Visualization, Writing – original draft, Writing – review & editing. WG: Conceptualization, Data curation, Formal analysis, Funding acquisition, Investigation, Methodology, Project administration, Resources, Software, Supervision, Validation, Visualization, Writing – original draft, Writing – review & editing. JY: Methodology, Conceptualization, Formal analysis, Project administration, Validation, Investigation, Visualization, Writing – original draft, Writing – review & editing. GF: Data curation, Methodology, Supervision, Conceptualization, Project administration, Validation, Funding acquisition, Resources, Visualization, Software, Writing – original draft, Writing – review & editing.
